# Nonsurgical Orthodontic Treatment of a Severe Open Bite Case Using Miniscrews with Modified Multiloop Edgewise Arch Wire Technique

**DOI:** 10.1155/2022/1844167

**Published:** 2022-09-15

**Authors:** Abdulkarim A. Hatrom, Bushra Kanwal, Fatima Hamooda, Hashim A. Alzahrani

**Affiliations:** ^1^Department of Orthodontics, Alnoor Hospital, Ministry of Health, Makkah, Saudi Arabia; ^2^Private Practitioner, AL Baha, Saudi Arabia; ^3^Private Practitioner, Mecca, Saudi Arabia; ^4^Orthodontics Division, Security Force Hospital, Mecca, Saudi Arabia

## Abstract

Skeletal open bite is one of the most challenging malocclusions to treat and maintain due to the difficulty and instability of correction. Although a combination of orthodontic treatment and orthognathic surgery may be the ideal approach in most cases, the complications, risks, and costs of surgery have sparked an interest in alternative treatment options that use temporary anchorage devices to achieve orthognathic-like effects. Adult patients can be treated without the need for special compliance using temporary anchorage devices such as miniscrews. This case report demonstrates a goal-oriented strategy for nonsurgical treatment of a complex skeletal open bite malocclusion in an adult patient using miniscrews and a modified multiloop edgewise arch wire (MEAW) technique, with the results evaluated clinically and cephalometrically.

## 1. Introduction

Anterior open bite (AOB) is defined as a condition in which there is no contact and no vertical overlap of the lower incisor crown with the upper incisor crown when the mandible is in full occlusion [1]. In an adult Caucasian American population, the prevalence of AOB was reported to be around 3%; however, it can range anywhere from 1.5 to 11 percent depending on the ethnic group and dental age [2]. The most common cause of a complex AOB malocclusion is a combination of habit, skeletal, dental, and functional effects [3]. Beyond the complexity of treatment and long-term stability of the open bite correction, it is considered a challenging treatment due to the high risk of vertical relapse, regardless of treatment technique or retention protocol [4]. Subsequently, a successful outcome requires a careful initial examination, diagnosis, treatment plan, and evaluation of habitual risk factors. A high level of patient compliance with retainer wear is also a crucial factor.

This case report reveals the nonsurgical and nonextraction orthodontic treatment of a 4 mm AOB malocclusion in a 21-year-old girl with a combination of miniscrews in the posterior maxillary area for intrusion and modified MEAW technique.

The MEAW approach, which consists of L-shaped loops between the teeth, was first described by Kim in 1987 [5]. Some authors have improved the MEAW approach by combining anterior vertical elastics with exaggerated curve Spee nickel-titanium (NiTi) arch wires for maxillary teeth and reverse curve Spee NiTi arch wires for mandibular teeth [6]. However, the success of treatment depends largely on patient compliance in wearing intermaxillary elastics.

## 2. Diagnosis and Treatment Planning

**Figure 1:**
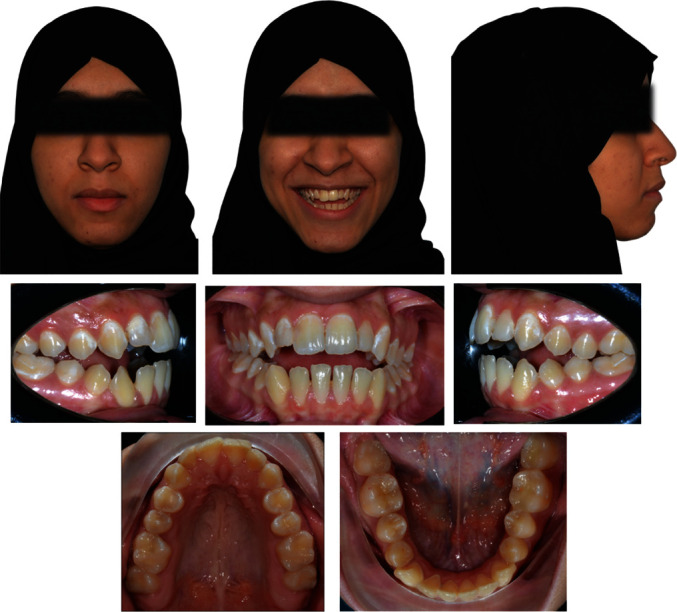
Pretreatment extraoral and intraoral photographs of the patient.

**Figure 2:**
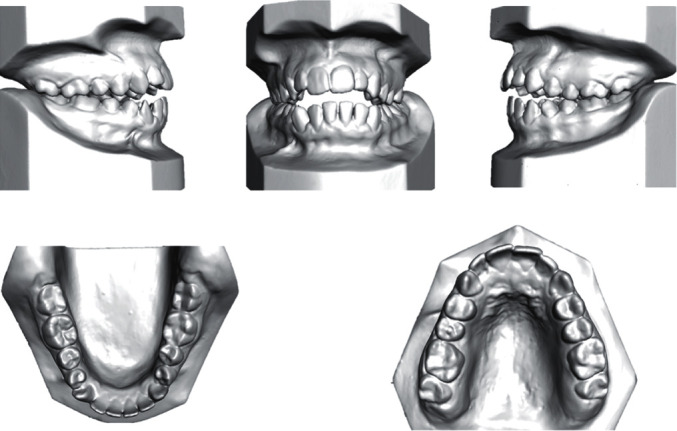
Pretreatment models.

A female patient aged 21 years presented to the Orthodontic Department of Security Force Hospital with a chief complaint of gaps between the upper and lower anterior teeth. She had no significant medical or dental history. Clinical extraoral examination showed a symmetrical face, convex profile, and competent lips. Smile analysis found a flat smile arc. The lower dental midline was shifted 2 mm to the left of the mid-sagittal plane. Intraorally, she had a class I molar relation on the left side and class III on the right side. She had an overjet of 3 mm and a symmetric anterior open bite of 4 mm extending from the premolar to premolar. The lower anterior teeth showed a mild amount of spacing. The spacing was measured using the arch perimeter analysis where the length of the arch measured using a brass wire was subtracted from the total mediodistal width of the teeth mesial to the first permanent molars. During functional evaluation, a forward and adaptable tongue posture was identified, which was visible during speech and swallowing. The posture of the tongue was evaluated in the rest position and during function. The patient's mouth remained slightly open at rest and the tongue rested between the upper and lower anterior teeth. During function, the patient thrusted the tongue to achieve an oral seal while swallowing. During the pronunciation of the sibilant sounds [s] and [z], the patient's speech was affected (Figures 1 and 2).

**Figure 3:**
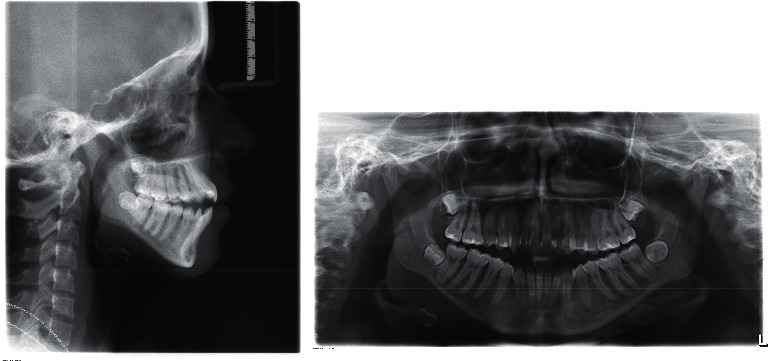
Pretreatment cephalometric and panoramic radiograph.

Cephalometric analysis showed a Class I skeletal relationship (ANB angle = 3°), a steep mandibular plane angle (GoG-SN = 38.2°) and increased maxillary-mandibular plane angle (MMPA = 34.7°). On dental analysis, she had mildly proclined upper incisors but still within the normal range (U1-PP = 115°) and lower incisors within the normal range (L1-MP = 89.2°) (Figure 3).

The panoramic radiograph showed all complement of teeth with no noticeable root resorption. Tooth 38 showed signs of impaction. No caries or periapical lesions were seen (Figure 3).

### 2.1. Treatment Objectives

The main objective of orthodontic treatment was to eliminate the anterior open bite while achieving satisfactory smile aesthetics and masticatory function. To provide stability in the treatment results, the elimination of the abnormal tongue posture was proposed during the active phase of treatment.

### 2.2. Treatment Options

A combined orthodontic surgical approach was the first treatment option presented to the patient. The open bite would be corrected by posterior maxillary impaction, followed by consequent mandibular autorotation [7].

The extraction of the first molars or premolars, followed by anterior retraction and mesialization of the posterior teeth during space closure, was a second treatment option. The uprighting and extrusion of the incisors, as well as the counterclockwise rotation of the mandible, would help to correct an open bite [8].

The use of intermaxillary elastics to extrude the anterior teeth was proposed as a third approach for managing the open bite. This option was anticipated to have little or no skeletal effect, and it would necessitate a lot of dental extrusions and patient compliance [9].

A fourth option was to use skeletal anchorage in conjunction with the modified MEAW technique to intrude the maxillary molars with some extrusion to enhance the smile and gingival display [9–12]. While the open bite was being addressed, this alternative had the potential to give minimal positive facial changes.

The patient refused surgery due to the impact it would have on her appearance. The second option was ruled out due to the possibility of aggravating the patient's lip retrusion during the anterior teeth retraction. Since the third option is prone to relapse and excessive gingival display, it was also excluded. Even though the fourth option has the most biomechanical difficulties, it believes to produce the best skeletal and dental results.

### 2.3. Treatment Progress

**Figure 4:**
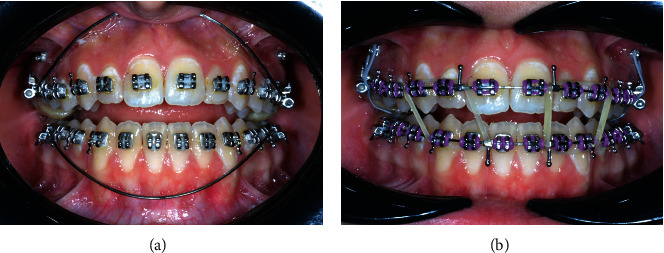
(a) Passive NiTi curved arches; and (b) anterior vertical elastic.

**Figure 5:**
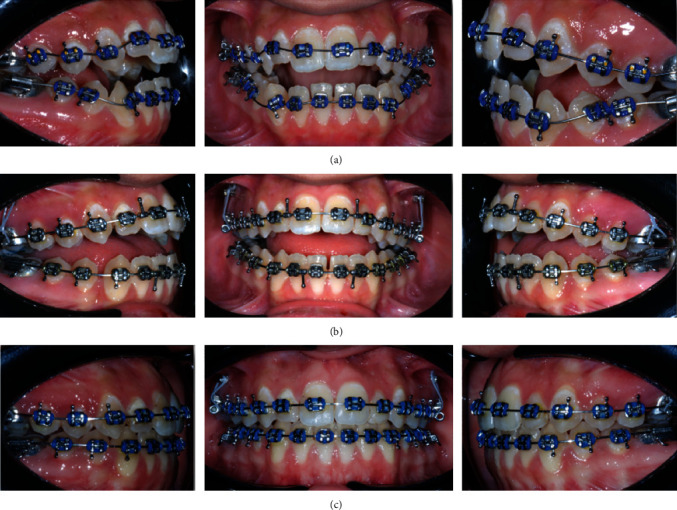
(a, b, and c) Progress photographs.

Treatment Sequence and Biomechanical planning is shown in Table 1. The fixed appliances consisted of preadjusted brackets following a Roth 0.018-inch prescription (3M Unitek, Monrovia, California, USA). A sequence of 0.014, 0.016, 0.016 × 0.022 inch NiTi arch wires (3M Unitek) were engaged for initial leveling and alignment following which, a 0.017 × 0.025 inch NiTi arch wire with accentuated curve of Spee in the maxillary arch and reverse curve of Spee in the mandibular arch was used with anterior box elastics ¼ inch measured with a gauge (Dentaurum, Ispringen, Germany); at the rest position, the forces were standardized to 100 g per side. Crimpable hooks were placed between the upper and lower incisors to facilitate elastic wear (Figure 4). Four 8 mm × 1.8 mm miniscrews (3M Unitek), two buccal and two palatal were inserted between the second premolar and first maxillary molar buccally and between the first maxillary molar and second maxillary molar palatally to start posterior intrusion mechanics via miniscrews (Figure 5) Short elastic chain (3M Unitek) was used to apply the intrusion forces. The second molars were intruded along with the first molars by means of a rigid segmental stainless-steel wire bonded occlusally from the first molar to the second molar on both sides. Considerable reduction of the anterior open bite had occurred after 4 months of starting molar intrusion. The maxillary and mandibular interdental spaces were closed by elastic chains acting from the first molar to the first molar.

The fixed appliances were removed after 20 months of active treatment. Vacuum-formed retainers in the upper and lower arch along with a fixed lingual retainer were bonded in the lower arch.

### 2.4. Treatment Results

**Figure 6:**
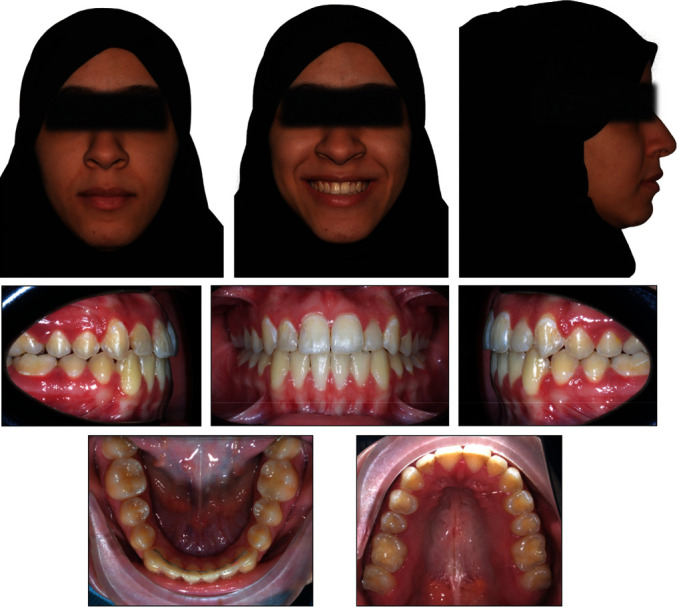
Posttreatment photographs of the patient.

**Figure 7:**
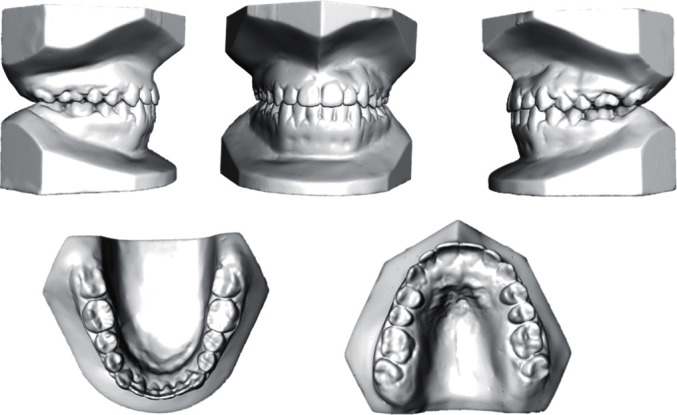
Posttreatment models.

**Figure 8:**
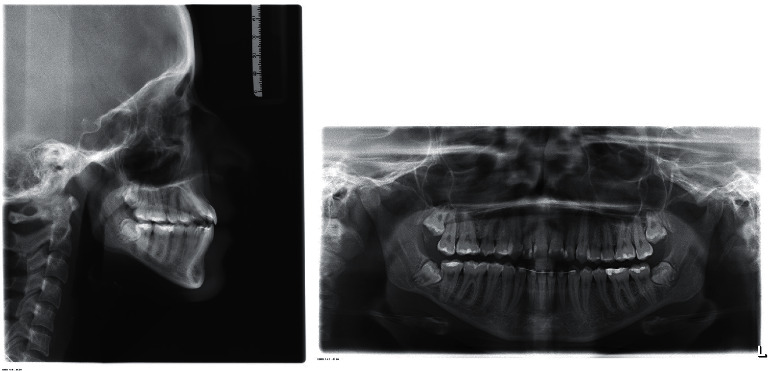
Posttreatment cephalometric and panoramic radiograph.

**Figure 9:**
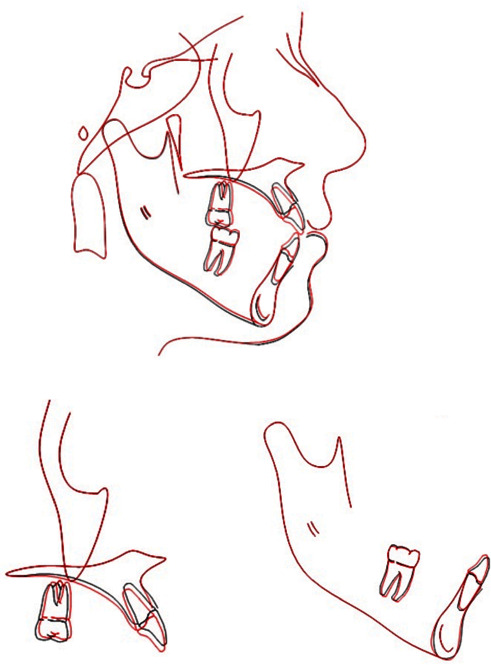
Posttreatment cephalometric superimposition.

A harmonious facial balance, improved smile, and good interdigitation were achieved at the end of treatment (Figure 6). The intraoral photographs and cephalometric analysis showed that there was a 6 mm improvement in the overbite, from −4 to 2 mm, an increase in mandibular protrusion (SNB angle) from 80° to 81.9°, as a result of the counterclockwise rotation. The maxillary molars were intruded by 1.9 mm and maxillary incisors extruded by 1 mm. The lower incisors extruded by 0. 7 mm. There was no obvious evidence of clinically significant root resorption or dental caries throughout the treatment. The third molars showed signs of impaction and were advised for prophylactic removal (Figures 7–9).

## 3. Discussion

In nongrowing patients generally, extrusion of the posterior teeth accompanies an anterior open bite. Intruding or inhibiting the vertical movement of the posterior teeth is the optimal treatment strategy. The patient's balanced facial appearance, profile, and the risk involved in surgery influenced in making the decision to use nonsurgical options with skeletal anchorage combined with the modified MEAW technique, which has been shown to be beneficial in treating open bite malocclusions [6]. Briefly, the modified MEAW technique uses NiTi arch wires as an accentuated curve of Spee for the maxillary arch and a reverse curve of Spee for the mandibular arch to correct the open bite [6]. The anterior vertical elastics used in this technique transfer the intrusion effect of the curved arches on the anterior teeth to the posterior region to control the vertical position of the posterior teeth. Thus, the open bite is closed by uprighting the posterior teeth, extruding and retracting the anterior teeth, and changing the maxillary and mandibular occlusal planes' inclinations toward each other [6]. However, it has been noted that this technique necessitates a high level of expertise [13].

The correction of the anterior open-bite resulted mostly from a counterclockwise rotation of the mandible (caused by the intrusion of the maxillary posterior teeth). Excessive extrusion of the incisors to close an anterior open bite is not recommended since the condition will have a high risk of relapse once the appliances are removed [14]; however, slight extrusion was done in this case to enhance the gingival display and smile. Treatment should instead aim to intrude on the molars or partially restrict their vertical development. The amount of miniscrews molar intrusion was 1.9 mm and is similar to the average intrusion previously reported [12, 15]. Temporary anchoring devices (TADs), which have a significant clinical value, offer skeletal anchorage for effective orthodontic treatment and are widely employed in the orthodontic field. Miniscrews have a number of benefits, including minimal cost, easy placement and removal, and sufficient anchorage to support tooth movement [16]. Concerning stability and patient safety, complications can occur during miniscrews placement and following orthodontic loading. Although complicated malocclusions are more frequently treated with miniscrews [17], root damage and other problems are possible [18]. Therefore, throughout treatment planning, these concerns should be thoroughly assessed and discussed with patients.

The stability of the corrected open bite in the current case report needs to be closely monitored [19]. However, there was less than 0.5 mm relapse over 2 years in cases treated with Kim Mechanics where the overbite increased by 4 mm during treatment which is similar to the treatment described in the present case report [20]. But there are contrasting studies where more than 35% of the patients Lopez-Gavito et al. treated with an anterior open bite in the postretention phase experienced treatment relapse [21]. Remmers et al. examined 52 patients who had an open bite before treatment and found that 27% of patients who had successful treatment still had open bites five years following treatment [22]. Despite using a different criterion for overbite assessment, Jonson et al. study revealed negative overlap in 25.8% of the samples at the completion of the posttreatment period [9]. Considering the varied results for the stability of open bite correction, we could have achieved overcorrection of the overbite in the current case report to prevent any relapse.

Unfavorable tongue posture and inappropriate orthodontic tooth movement, such as incisor extrusion, may also contribute to relapse in corrected open bite cases.

In the present case report, the patient thrusted the tongue forward to contact with the lips to form an anterior seal that was adaptive to the underlying malocclusion. When the treatment was complete, and a positive overbite was achieved the tongue thrust ceased. The patient was taught tongue training exercises during the course of treatment to correct the tongue posture and periodically recalled to monitor the recurrence of the tongue thrusting habit posttreatment.

In this case report, the GoGn to Sn angle decreased by 2°. The alterations in overbite following treatment have a strong and positive connection with the pretreatment SN-GoGn angle, according to Beckman et al. [23]. Only the mandibular and palatal plane angles at the start of the treatment revealed a significant association with posttreatment overbite alterations among several variables [22]. However, the researchers claimed that as this was only possible by chance, the open bite could not be accurately predicted using pretreatment cephalometric factors. There are no specific cephalometric markers that can anticipate difficult cases. Additionally, there was no correlation between overbite at the start of treatment or changes to it throughout treatment with the frequency of treatment relapses, according to the study by de Freitas et al. [8]. In a research by Dung and Smith that looked at 300 participants, the overbite depth indicator (ODI) and the extent of anterior open bite at the beginning of treatment were the best predictors of the outcome [24]. Future research should explore assessing the effects of growth, posttreatment aspects such as the use of retainers, patient cooperation, and extending the follow-up period, as well as conducting prospective studies to assess the stability of open bite correction.

## 4. Conclusion

The nonsurgical orthodontic treatment of an adult patient with a complex anterior open bite using a mix of fixed appliances, vertical intermaxillary elastics, and skeletal anchorage for upper molar intrusion is described in the current case report. Considering the risks, patients with an anterior open bite may be successfully treated for molar intrusion by using miniscrews as anchorage. To achieve the correction, the patients' cooperation in wearing elastics was crucial. However, to guarantee long-term stability, follow-up visits are required.

## Figures and Tables

**Table 1:** Treatment sequence and biomechanical plan.

Maxilla	Mandible
Band molars, bond maxillary arch, and start leveling with 0.014, 0.016, 0.016 × 0.022 inch NiTi arch wires	Band molars, bond mandibular arch, and start leveling with 0.014, 0.016, 0.016 × 0.022 inch NiTi arch wires
Progress to 0.017 × 0.025 inch ACS Niti	Progress 0.017 × 0.025 inch RCS Niti
Surgical hook between the central and lateral incisors right and left was crumbed	Surgical hook between the central and lateral incisors right and left was crumbed
Occlusal splint between upper 6 and 7 in both sides was cemented	
TAD inserted between upper 6 and 5 buccally and 6 and 7 palatally in both sides and started intrusion using an elastic chin	
Box elastic ¼ inch, 3.5 oz from the surgical hook and canine in the upper arch to the surgical hook and canine in the lower arch on both sides	
Continue with 0.017 × 0.025 inch ACS NiTi arch wire and box elastic ¼ inch, 3.5 oz from the surgical hook and canine in the upper arch to the surgical hook and canine in the lower arch in both sides	Continue with 0.017 × 0.025 inch RCS NiTi arch wire
Progress to 0.016 × 0.022 inch SS with open elastic chine from 6 to 6 to close spaces	Progress to 0.016 × 0.022 inch SS with open elastic chine from 6 to 6 to close spaces
Continue using elastic chine for intrusion	
Continue with 0.016 × 0.022 inch SS with finishing bends	Continue with 0.016 × 0.022 inch SS with finishing bends
Debond and vacuum-formed retainer	Debond and fixed lingual retainer from 3–3 with a vacuum-formed retainer
Six months recall appointment for retention check	Six months recall appointment for retention check

ACS: accentuated reverse curve of Spee, NiTi: nickel-titanium, RCS: reverse curve of Spee, TAD: temporary anchorage device, SS: stainless steel.

## References

[1] Gu D., Leroux B., Finkleman S. (2022). Anterior openbite malocclusion in adults. *The Angle Orthodontist*.

[2] Bilgic F., Gelgor I. E., Celebi A. A. (2015). Malocclusion prevalence and orthodontic treatment need in central Anatolian adolescents compared to European and other nations’ adolescents. *Dental Press Journal of Orthodontics*.

[3] Stojanovic L. (2007). Etiological aspects of anterior open bite. *Medicinski Pregled*.

[4] Cambiano A. O., Janson G., Lorenzoni D. C., Garib D. G., Dávalos D. T. (2018). Nonsurgical treatment and stability of an adult with a severe anterior open-bite malocclusion. *Journal of Orthodontic Science*.

[5] Kim Y. H. (1987). Anterior openbite and its treatment with multiloop edgewise archwire. *The Angle Orthodontist*.

[6] Erdem B., Kucukkeles N. (2018). Three-dimensional evaluation of open-bite patients treated with anterior elastics and curved archwires. *American Journal of Orthodontics and Dentofacial Orthopedics*.

[7] Maia F. A., Janson G., Barros S. E., Maia N. G., Chiqueto K., Nakamura A. Y. (2010). Long-term stability of surgical-orthodontic open-bite correction. *American Journal of Orthodontics and Dentofacial Orthopedics*.

[8] de Freitas M. R., Beltrão R. T. S., Janson G., Henriques J. F. C., Cançado R. H. (2004). Long-term stability of anterior open bite extraction treatment in the permanent dentition. *American Journal of Orthodontics and Dentofacial Orthopedics*.

[9] Janson G., Valarelli F. P., Henriques J. F. C., de Freitas M. R., Cançado R. H. (2003). Stability of anterior open bite nonextraction treatment in the permanent dentition. *American Journal of Orthodontics and Dentofacial Orthopedics*.

[10] Baek M. S., Choi Y. J., Yu H. S., Lee K. J., Kwak J., Park Y. C. (2010). Long-term stability of anterior open-bite treatment by intrusion of maxillary posterior teeth. *American Journal of Orthodontics and Dentofacial Orthopedics*.

[11] Deguchi T., Kurosaka H., Oikawa H. (2011). Comparison of orthodontic treatment outcomes in adults with skeletal open bite between conventional edgewise treatment and implant-anchored orthodontics. *American Journal of Orthodontics and Dentofacial Orthopedics*.

[12] Hart T. R., Cousley R. R. J., Fishman L. S., Tallents R. H. (2015). Dentoskeletal changes following mini-implant molar intrusion in anterior open bite patients. *The Angle Orthodontist*.

[13] Ribeiro G. L., Regis S., da Cunha T. . M. A., Sabatoski M. A., Guariza-Filho O., Tanaka O. M. (2010). Multiloop edgewise archwire in the treatment of a patient with an anterior open bite and a long face. *American Journal of Orthodontics and Dentofacial Orthopedics*.

[14] Sugawara J., Aymach Z., Nagasaka H., Kawamura H., Nanda R. (2011). Non-surgical correction of skeletal open bite: a goal-oriented approach evaluated by CBCT. *Journal of Clinical Orthodontics*.

[15] Xun C., Zeng X., Wang X. (2007). Microscrew anchorage in skeletal anterior open-bite treatment. *The Angle Orthodontist*.

[16] Kravitz N. D., Kusnoto B. (2007). Risks and complications of orthodontic miniscrews. *American Journal of Orthodontics and Dentofacial Orthopedics*.

[17] Alogaibi Y. A., Afify A. R., Al-Fraidi A. A., Hassan A. A. (2020). Nonsurgical treatment of class III malocclusion with both anterior and posterior Crossbites combined with impacted and congenitally missed teeth. *Case Reports in Dentistry*.

[18] Montasser M. A., Scribante A. (2022). Root injury during interradicular insertion is the most common complication associated with orthodontic miniscrews. *The Journal of Evidence-Based Dental Practice*.

[19] Greenlee G. M., Huang G. J., Chen S. S. H., Chen J., Koepsell T., Hujoel P. (2011). Stability of treatment for anterior open-bite malocclusion: a meta-analysis. *American Journal of Orthodontics and Dentofacial Orthopedics*.

[20] Kim Y. H., Han U. K., Lim D. D., Serraon M. L. P. (2000). Stability of anterior openbite correction with multiloop edgewise archwire therapy: a cephalometric follow-up study. *American Journal of Orthodontics and Dentofacial Orthopedics*.

[21] Zuroff J. P., Chen S. H., Shapiro P. A., Little R. M., Joondeph D. R., Huang G. J. (2010). Orthodontic treatment of anterior open-bite malocclusion: stability 10 years postretention. *American Journal of Orthodontics and Dentofacial Orthopedics*.

[22] Remmers D., van’t Hullenaar R., Bronkhorst E. M., Bergé S. J., Katsaros C. (2008). Treatment results and long-term stability of anterior open bite malocclusion. *Orthodontics & Craniofacial Research*.

[23] Beckmann S. H., Segner D. (2002). Changes in alveolar morphology during open bite treatment and prediction of treatment result. *European Journal of Orthodontics*.

[24] Dung D. J., Smith R. J. (1988). Cephalometric and clinical diagnoses of open bite tendency. *American Journal of Orthodontics and Dentofacial Orthopedics*.

